# Case of Polypus Uteri, Successfully Removed by Ligature and Excision

**Published:** 1840-05-02

**Authors:** Jacob G. Walker


### CASE OF POLYPUS UTERI, *Successfully removed by ligature and excision.* By JACOB G. WALKER, M. D.

To the Editors of the Medical Examiner.

*Leasburg, Carswell Co. (N. C.)* April 24, 1840.

GENTLEMEN:—The following case of uterine polypus, extraordinary for its magnitude, (weighing four pounds, eight ounces,) is communicated to you, for insertion in your valuable periodical, which, if you think worthy of such, is at your disposal.

Yours, very respectfully,
JACOB G. WALKER.

Mrs. S——, the mother of five children, the youngest of which, about four years old. Some time after its birth, she experienced more or less symptoms of local irritation in the neighbourhood of the womb, unaccompanied for a considerable period with any signs of tumefaction or visible manifestations of disease. Ultimately, however, about two years since, (or the rise,) there appeared upon examination, by Dr. J. Comer, (then her physician in charge,) an oval tumour, the size apparently of a hen's egg, who, upon continuing his examination, (per vaginam,) suspected the existence of polypus, and upon disclosing the same, and the probable necessity of committing her case to the doubtful, but sometimes efficient operations of nature, desisted from further treatment than that of a mere palliative one. But after the lapse of some months, the rapid growth and augmentation of the tumour, and consequent distention and irritation communicated to the uterine vessels, had the effect of exciting periodical hæmorrhages at intervals, recurring every two or three weeks, which, for the time, would soften and relax the tumour, immensely, accompanied with symptoms of great prostration and exhaustion, peculiar to sanguineous discharges.

This course of hæmorrhagic visitations, has been kept up (by the phenomena and operations of nature) pretty much for the last twelve or eighteen months, during which time the writer of this article has had frequent opportunities of witnessing and examining the various stages of growth, in the development of this frightful and malignant disease; and has no doubt, but that the growth has been greatly retarded in consequence of the frequent losses of blood, notwithstanding they have had the effect of greatly impairing and undermining the patient's constitution.

However, about two or three months previous to the removal of this tumour, there was a suspension of hæmorrhagic discharges, succeeded by an increased rapidity in growth, to that degree, that the uterine cavity and vaginal canal were literally blocked up with partial protrusion beyond the os externum, the abdominal tumefaction almost equalling that in the last stage of utero-gestation, until, on the night of the 14th instant, she was suddenly attacked with the usual symptoms of labour-pains, with unusual violence, which continued unabatingly until I was sent for; and found upon arrival, that the tumour had presented near one-third; and, from its distension, there was a complete suppression in the urinary and fœcal discharges, while in attempting to introduce the catheter, it was impracticable to find the mouth of the urethra, and the duration of the urinary suppression, the consequent distension of the bladder, contributed much to the aggravation of existing symptoms.

Under these circumstances, the entire suppression of urine, the impracticability of drawing it off, being unable to push back the mass, we concluded that there was no alternative but death, without the removal of this fungous growth; therefore, Dr. J. Comer was called in, who agreed with me as to the propriety of undertaking the operation, which consisted in a gentle but persevering traction at the protruded portion, co-operative with the uterine contractions, which were very active at this period, until at length we succeeded in delivering the principal bulk of the tumour, clear of the vagina and os externum; finding it impossible to advance any farther, we determined on applying the ligature as high up the pedicle as possible, after which, we excised the tumour below the ligature, and subjected the same to weight, which proved to weigh four pounds eight ounces, or perhaps five pounds, including the neck.

The woman is, at the above date, apparently doing well, and advancing towards convalescence.

